# Integrations between Autonomous Systems and Modern Computing Techniques: A Mini Review

**DOI:** 10.3390/s19183897

**Published:** 2019-09-10

**Authors:** Jerry Chen, Maysam Abbod, Jiann-Shing Shieh

**Affiliations:** 1 Department of Mechanical engineering, Yuan Ze University, Taoyuan 32003, Taiwan; 2 Department of Electronic and Computer Engineering, Brunel University London, Uxbridge UB8 3PH, UK

**Keywords:** autonomous, intelligent control system, machine learning, IoT, big data, federated learning

## Abstract

The emulation of human behavior for autonomous problem solving has been an interdisciplinary field of research. Generally, classical control systems are used for static environments, where external disturbances and changes in internal parameters can be fully modulated before or neglected during operation. However, classical control systems are inadequate at addressing environmental uncertainty. By contrast, autonomous systems, which were first studied in the field of control systems, can be applied in an unknown environment. This paper summarizes the state of the art autonomous systems by first discussing the definition, modeling, and system structure of autonomous systems and then providing a perspective on how autonomous systems can be integrated with advanced resources (e.g., the Internet of Things, big data, Over-the-Air, and federated learning). Finally, what comes after reaching full autonomy is briefly discussed.

## 1. Introduction

In 1950, Turing asked “Can machines think?” This simple question has sparked a decades-long debate in the artificial intelligence (AI) community [1]. Turing formulated the well-known Turing test (TT) or imitation game to answer this question. The TT requires at least three players to run: One interrogator played by a human and two other interrogated “persons” played by a human and machine. The interrogator’s job is to identify which of the interrogated “persons” is human by asking a series of questions, and the interrogated “person’s” responsibility is to convince the interrogator that they are the only true human [2,3]. Although the TT was initially meant to test whether a machine has intelligence, the rules and perception of the test have varied during the following decades (along with changing attitudes toward AI in the scientific community) [4]. Issues [1] surrounding the rules of TT have also affected this changing attitude. For example, because the machine is judged by how well it imitates something it is not, perhaps it would be fair that the machine is compared against a person who is also imitating something he/she is not [5,6]. Nonetheless, although it is debatable whether machines can “think,” the fact that machines can learn is definitely not.

**Figure 1:**
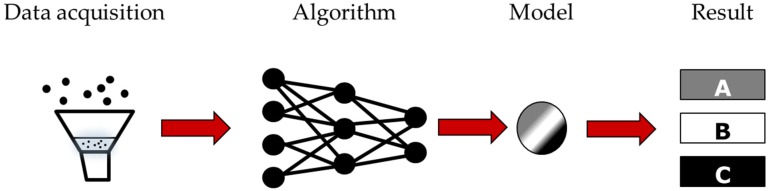
The flow chart of learning base.

The learning-based algorithm was first conceptualized in the mid-20th century [7], a mere few years after the conceptualization of neural network architectures [8]. Thereafter, the structure and algorithms for machine learning have been continuously developed [9]. With wider implementation of AI and the increasingly complex tasks that machines can perform in many fields, such as machine health monitoring [10], visual understanding [11,12], and bio-informatics [13], machines have exhibited an outstanding ability to learn, even surpassing humans in some tasks [14]. Every learning process begins by data acquisition, where the original data contain the key features of the given task. The machine then uses a selected algorithm to generate the corresponding model. The model is then adopted by the machine as a guideline for appropriate action (Figure 1). Once the best model has been chosen and constructed, the machine, in theory, will consistently obey the instructions of the model.

However, in real situations, environmental uncertainty may decrease the model’s predictive accuracy even if the model itself has not malfunctioned. One reason for this is that the uncertainty results in a data input to the model that is different from those previously used in the training. Such data are excluded by either removing the contaminations manually (which restores the system back to its initial state) or retraining the model with the present data. However, although such present uncertainty can be accounted for after several corrections, but future uncertainty requires the reactivation of such a learning process. Once the machine has finished training, the parameters it relies on to act are fixed (at least relative to the model). Thus, after decades of development in AI, we have only taught the machine to “learn” but not to “think.”

Thus, is it possible to develop a self-governing system for machines that adapts to its environment like a human being? The answer is hidden in the implementation of autonomy in engineering, where the eagerness to confront the inevitability of uncertainty drives the quest for an autonomous system.

## 2. Autonomy in Engineering

**Figure 2:**
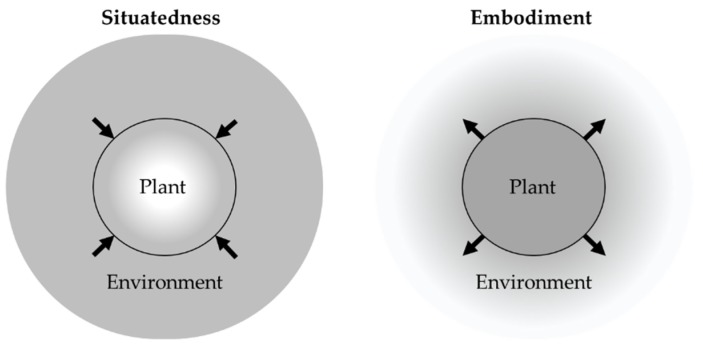
Interactions between an autonomous plant and its environment. A situated plant is affected by its environment, and an embodiment plant is part of the dynamic of the environment.

“Autonomy” is self-governance (whether of a polity or an individual). Etymologically in Greek, *auto* is “self” and *nomos* is “law” or “custom.” This profound concept [15] is of great philosophical interest with regard to the relationship between free will and moral responsibility [16]. However, in the field of engineering, “autonomous” is used as an adjective to describe systems having the ability of self-governance [17]. More precisely, according to [18], “a system is autonomous, with respect to a set of goals and under a set of uncertainties, if it achieves these goals under those uncertainties without external intervention.” Autonomous systems behave like human beings and perform complex tasks under varying degrees of uncertainty. To be able to address the uncertainty from environmental disturbances, autonomous systems must be able to sense and interact with their environment. Such an ability is being implemented [19] with two characteristics: situatedness and embodiment. Situatedness means that the “here” and “now” of the environment directly influence system behavior, where the system deals with the world it is situated in rather than with abstract descriptions. Embodiment implies some mechanism that allows a system to interact with its surroundings, where actions of the system are part of the dynamic of the environment. An example of systems that are situated but not embodied are monitoring systems, where the environment affects the system but not vice versa. By contrast, an example of an embodiment-only system is the traditional industrial robot on production lines. Its movement is pre-programmed before operation—and is thus capable of interfering in the environment—but its operation is not guided by the current state of its environment. Figure 2 illustrates the opposite direction of the system-environment interaction of these two features. This distinction was used in [20] for distinguishing mobile robots from computer simulations.

To make problem solving more human-like, researchers have listed various capabilities an autonomous system must have [21] and have provided a taxonomy of autonomous agents [22]. Although these definitions differ in terms of their features, they essentially demand that autonomous systems should be independent. Specifically, an independent autonomous system is able to face environmental uncertainty without intervention from an operator. Because of this demand, the system must be constructed with a hierarchical structure that can manage the execution and monitoring of the actions required to meet its goals. Environmental uncertainty can be used to model the level of autonomy required by an autonomous system, and it is useful for improving the design of autonomous systems for resolving environmental uncertainty. To independently complete a task, autonomous systems should be able to adapt to their environmental changes (whether caused by external interference or the internal variations of parameters). Although autonomous systems are well-developed in robotics [23] and self-driving vehicles [24,25], applications in biomedical fields are rare. However, the concept of an autonomous system is perfectly suited to medical applications, promising substantial improvements in the reliability of long-distance medical transportation and medical care in remote areas. To achieve the autonomy, the autonomous system must have at least the functions and structure proposed in Section 2.2. The design of these functions and structures derive from the essential features of autonomous systems that are described in the following subsection.

### 2.1. Features of Autonomous System

#### 2.1.1. Independency

Independence is important because of uncertainty. In any industry, an independent autonomous control system promises labor-hour savings in its operation. With their ability to adapt, such systems can self-monitor and solve problems during operation. Independence is also a new goal in modern engineering. Previously, the aim was to centralize the production line by having it controlled by a single computer. However, the current aim, made possible by advances in AI and microprocessor design, is to decentralize the governance of machines, allowing each to be self-governing (e.g., in edge computing). The direction of human evolution points toward doing more with less. In early civilization, animal power (e.g., in agriculture and transportation) replaced human power, which then evolved into the use of machines. Similarly, humanity has been searching for a replacement to human intelligence. This shift from human intelligence to computer intelligence is observed not only in AI but also in control systems. In fact, AI has been directly used as a type of control system (e.g., in neural networks). This shift in intelligence also appears in intelligent control, which, unlike classical control, transfers the workload from the system designer to the system itself [26].

However, the self-governance and independence that are required for autonomous control systems increase only their autonomation but not automation. Derived from the basic concepts described in [27], which provides a discussion on the differences between biological and robotic autonomy, automation and autonomation differ in terms of the essence of different forms of autonomy. In automation, the operator expends less “effort” to monitor and calibrate the behavior of the system. By contrast, in autonomation, the operator has a diminished “ability” to intervene in the intention and behavior of the system. Therefore, autonomous control systems should be independent for the sake of automation but not autonomation. Specifically, irregardless of how independent an autonomous system is, it must be able to cooperate with other systems and be assessed by its supervisor or some higher-level system. Having the ability to switch between cooperative and independent action widens the operating range of an autonomous system.

#### 2.1.2. Wide Operating Range

A wide operating range of an autonomous control system enables its high tolerance for uncertainty, which is necessary when facing external disturbances. The wider the operating range, the more stable the autonomous control system. Autonomous control systems were initially intended for the optimization of (underwater, ground, air, and space) vehicles. For example, one study increased the operating regions of the control system by adding subsystems [28]. When given the instruction to maintain a vehicle’s speed, a simple system controlled only the accelerator and was thus suitable only for roads without obstacles. However, after merging with other subsystems (e.g., computer vision), the automobile could maintain its speed while dodging obstacles on the road, thus broadening its operating range. 

#### 2.1.3. Well-Identified Goals

Autonomy is uninteresting in the absence of a goal. Because a machine relies on the error of an assigned value to take action or adapt to its environment, goals define the mathematical model of the system that is abstractly constructed by the control system. Furthermore, the priority of goals is critical for constructing the model of the control system. Because most of the operating range of the autonomous control system is being restricted by the “safe” manner in which it is bounded to a higher-level goal, the goals and its priority must be clarified for an autonomous control system. In the previous example, the goal of maintaining vehicle speed was subordinate to the goal of passenger safety.

#### 2.1.4. Adaptation to Uncertainty

The essence of an autonomous control system is its ability to address uncertainty. Uncertainty exists because of imperfect knowledge: A system cannot be completely modeled, and an environment cannot be completely known (relative to a set of goals). The purpose of autonomy is the achievement of goals under environmental uncertainty. Specifically, goals are necessary because they dictate how a system adapts to its changing and uncertain environment. The larger the range of uncertainties that a system can resolve and the wider its operating range, the lesser the need for external intervention (whether by humans or superior systems) in its achievement of its goals, entailing a more autonomous system.

### 2.2. Autonomous System Structure

“*Every autonomous system is a control system*” quoted in [18], and it implies the autonomous system would always have a set of goals to be achieved and a control mechanism to achieve them. It is believed that the autonomous system may be evolved from existing control system. The basic idea of a control mechanism is using sensors for measurements and actuators to implement the decisions for feedback control. However, an autonomous system that can address uncertainty is a system that can adapt without human aid. The control mechanism of an autonomous system is more complex compared to other control systems. Fortunately, the structure proposed in [29] has concluded the fundamental mechanism of the autonomous system based on control mechanisms, and it relates to the following functions (arranged from low- to high-level).

1. Self-regulating: With a basic mechanism, the machine is able to operate continuously to fulfill a repetitive task. Because machines must be able to work continuously, this function is required.
2. Self-adapting: The system applies models for calibrations to optimize the process.
3. Self-organizing: The system is able to evaluate the implemented model or process to ensure that the system is optimized in relation to its goal.
4. Self-repairing: The system is capable of fixing problems in its internal parameters without external intervention. It is also able to report errors to other coherent systems.
5. Self-governing: The system can directly interact with any subsystem. Having the highest authority over its subsystems, it manages lower-level functions to reach its goals. This is the highest-level function.

**Figure 3:**
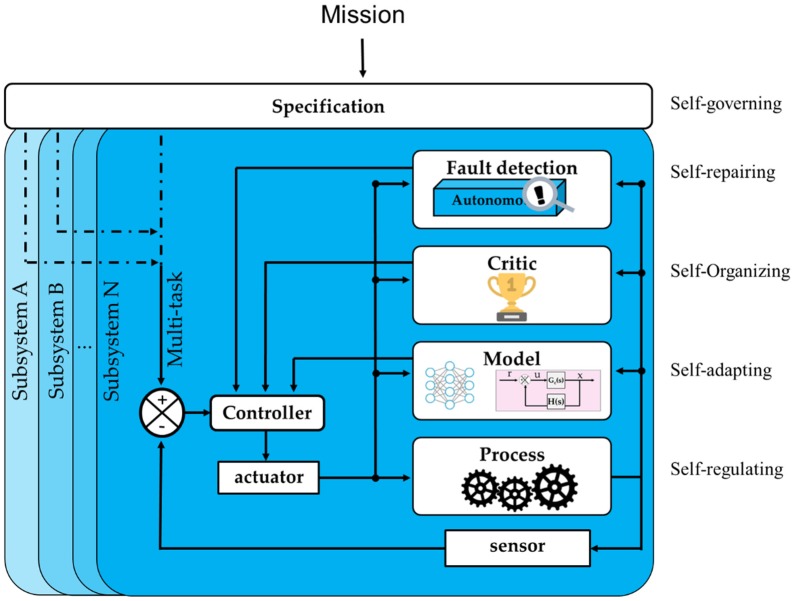
The autonomous system structure.

The general structure of an autonomous system relative to these functions is illustrated in Figure 3. Upon receiving its mission from a supervisor, the autonomous system first divides the mission into single specified tasks and subsequently apportions the tasks to its subsystems. The autonomous system then behaves according to the following structure to complete its given task.

#### 2.2.1. Actuator and Sensor

Uncertainty exists because of the impossibility of perfect knowledge of the environment and the available means (relative to a goal). Thus, to mitigate uncertainty, the system must have a sensor through which it obtains the varying parameters associated with the environment and its disturbances. Upon transmitting sensor-derived data to the controller, the higher-level system responds to these data according to the adopted algorithm. After several computations within the higher-level system, the result is eventually transmitted down the hierarchy. Finally, the result is interpreted by the execution level as an instruction or piece of information. The result that is being transmitted down the hierarchy does not necessarily go directly to the actuator because it could be information on strategy, thus corresponding to the subsystem’s task and its corresponding hierarchy level. However, if it is a demand for action, the subsystem should act accordingly by operating the actuator.

Sensors planted in the control system could be classify into two categories according to their functions, that are sensors installed for modeling the environment and sensors embedded for higher-level coordination. Sensors which are used to modeling the environment are crucial for the autonomous system because every decision that is made by the system is guided by the constructed “world” model. Thus, domain knowledge is highly relevant to the question of the types of data that are necessary for task completion. The world model is an autonomous system’s internal representation of the external world. According to [20,30,31], the world model comprises two types of information in the “environment model” and “world model.” The environment model describes the current state of the system’s surroundings, such as the spatial (3D) description of the physical objects in the environment. It contains dynamic and situational knowledge and is usually used for navigation tasks. By contrast, the world model includes more general knowledge about other possible states and the means to realize these states. It typically includes more invariant and general knowledge of events, processes, and the properties of and relations between objects.

Furthermore, sensors should be embedded in the system for higher-level coordination. For example, sensors embedded in the joint of a robot’s arm are responsible for higher-level coordination. They coordinate the movement of operations but do not attribute to the establishment of the world model. Such sensors are akin to sensors for torque measurement of the electrical, electromagnetic, and optical type [32]. Because of their small size and low cost, sensors such as microelectromechanical system inertial sensors are frequently used in navigation devices. With 3D accelerometers and 3D gyroscopes, inertial sensors can be integrated to obtain information on position and orientation quickly and at high sampling rates [33]. In fact, the function of sensors that are embedded in autonomous system is to not only replicate the physical environment or to convert physical stimuli into readable signals but also increase the precision and reliability of the system by simultaneously detecting and measuring the physical effect [34]. For example, the 3D gravitational transducer and magnetic compass that are mounted on the robot for Robot Haptic Control Interface are used to stabilize the direction of robot motion [35]. Nevertheless, sensors can also contribute to the critical and fault detection functions of the control system [36]. A fault is indicated if the residual between the actual and expected values exceeds a predefined threshold. Malfunctions can also be detected from the features of the computed characteristic quantities.

#### 2.2.2. Process

A process is a series of actions and decisions that enables a machine to achieve its goals. These actions and decisions are routines that comprise a prearranged set (and order) of executions from higher-level functions before each operation. The data generated and collected during the process contributes to higher-level modeling. The mechanism in the process is determined by the given task. In fact, the mechanism in the process is so crucial and specific it implies how the system is going to achieve its goals. The process function constructs the frames of control mechanism for other levels. The higher level depends on the data generated and collected in the process to develop models; the lower level relies on the process function to transform the control signal into action orders. The process is where the control system turns the orders and decisions computed by the model into practical actions. A well-organized process is also the cornerstone of intelligent manufacturing, as evident in applications in industry 4.0 [37]. With Internet of Things (IoT), cyber-physical systems (CPSs), cloud computing, big data analytics (BDA), and information and communications technology (ICT), the well-managed process enables intelligent manufacturing. 

Moreover, the process has the flexibility to be merged with other subsystems, sensors, and actuators through the IoT, as discussed in Section 4.1. Typically, every equipment that is being used in the production lines or other fields could be considered as the representation of process. It has embedded sensors to collect explicit data, a communication device to interact with a supreme system and actuators to perform the orders. In conclusion, this level of function performs tasks in two directions: From high to low level, practice the instructions; from low to high level, delivering data and information up the hierarchy.

#### 2.2.3. Model

The model is a mathematical object or an ad hoc specification of rules that is used to control the system or extract information from the system. Modeling algorithms have been well-developed in the fields of both classical and intelligent control systems. The selection of algorithms depends on its applicability. In practice, an intelligent control system is only used when classical control systems have failed [28]. For more details on how intelligent control can be applied to autonomous systems, please refer to [38]. In addition, the model of the system can also be implemented with machine learning and a neural network. With AI support, the autonomous system can complete more complex and abstract tasks; an example is given in Section 2.1.2.

The purpose of a model function is to extract information from the environment and apply it. Model function augments the system’s knowledge of the environment, where it includes more invariant and general knowledge about events, processes, and properties of and relationships between objects. Moreover, due to its rapid development, the model of the autonomous system can also be tested in virtual and augmented reality [39]. The software of the autonomous system that are being tested is mainly consisted of three phases—sense, plan, and act. The sense phase computes a perception of the environment based on incoming raw data to construct the world representations. Then the inner mechanism of the system generates a plan composed of actions based on the system’s current world representation, operational goals, and past experiences. Finally, the system executes the plan (which was made corresponding to the simulated situation) by sending the control signals to actuators. Then the simulated environment changes based on the interaction of actuators to complete the loop of the training model. In the initial phase of testing, the autonomous software can be tested through virtuality with a simulated hardware system and environment. Such virtual tests minimize risks and costs during model construction. Once the models become more stable, they can be introduced with more reality and less virtuality until physical testing. Before entering the final physical testing phase, augmented reality allows the autonomous system to be tested in an actual physical environment *sans* people and objects (which are projected with augmented reality) due to safety issues. One implementation is the Versatile Intelligent Portable Robot Platform (VIPRO) [40]. It allows the development of robot mechanical modeling in a 3D virtual environment, providing a virtual simulation platform for formulating strategies, developing robot motion, processing images, and facilitating the adaptive-intelligent and behavior-based control of robots. For example, the intelligent control interfaces that are integrated with VIPRO improves the performance of autonomous navigation robots [41].

#### 2.2.4. Critic Function

Uncertainty results in inconsistencies in the quality and quantity of data. This greatly affects the model because the data of the present state differ from those at the training stage. Moreover, with increasing data, the adopted model is less likely to be optimal. Thus, the autonomous system clearly requires a critic function for regular inspection of the model. The critic function is the quality control mechanism of the system: It maintains the optimal performance of the system under all conditions. Specifically, the critic function should change the current model when the model exhibits defects or when a better-performing model is available. An example of a human–robot (H2R) interaction system that has a critic function is detailed in [42]. The critic function uses reinforcement learning and neural networks to find the optimal parameters of the model to adjust the robot’s dynamics and minimize the tracking error. Other relevant algorithms for this function are genetic algorithms [43] and federated learning. Genetic algorithms implement Darwin’s principle of survival of the fittest, a characteristic of evolution, and federated learning is a form of distributed machine learning that will be discussed in Section 4.3.

#### 2.2.5. Fault Detection

Uncertainty is not only caused by external disturbances but also abnormalities in internal parameters or material fatigue. Thus, fault detection is indispensable for diagnosing system malfunctions. Fault detection in sensors [44] is especially crucial to an autonomous system because the modeling environment constitutes the fundamental element of every operation. This kind of mechanism have been the focus of studies of fault detection and diagnosis (FDD). For a relevant application of FDD in robotic systems, please refer to [45]. The actions in fault detection should have a three-stage conclusion. First, the system performs the error correction by automatically attempting to calibrate the parameter’s error. Second, if the error is not adjustable, the system should issue a report to a higher-level system to access the support of other repairing units. Third, if the situation is not containable and might damage the structure of the system, the system should be shut down by the authority command from a higher-level managing system.

#### 2.2.6. Specification

Specification governs the entire system including the subsystems. To improve system efficiency, the specification function must analyze the assigned mission and break it down into multiple tasks. Subsystems are not necessarily composed by all the pre-mentioned functions. Instead, the structure of one subsystem depends on its goals. Some sub-subsystems may only possess a certain degree of autonomous functions. Moreover, the specification function should possess the authority to rank the priority of the subsystems to prevent a subsystem’s operation from damaging the preferences. Specifically, every subsystem must be monitored to prevent the failure of single unit, which might cause the collapse of the entire structure of the system. Because this level of function requires high flexibility, it should be implemented with a control system that has excellent coordination, such as fuzzy control. Fuzzy control has been widely used in commercial products and applications. Moreover, a higher-level autonomous system entails a lower need for human intervention. In this case, fuzzy control has potential to perform this type of work. Please refer to [46,47] for more details on fuzzy control. Fuzzy logic enables the representation of uncertainty by allowing an input between 0 and 1, instead of the binary system of either 0 or 1. Thus, by applying fuzzy logic to intelligent decision support systems (including domain knowledge, modeling, and analysis systems) [48], decision support is extended by permitting the variables in the decision problem [49].

## 3. Hierarchy and Autonomy Levels

### 3.1. Hierarchy Level

For human-like behavior, autonomous control systems must have a hierarchical structure to manage the executions of the system with the assistance of a coordination interface [17]. Because human-like problem-solving techniques comprise the aim of autonomous control systems, every action taken by a control system should fit either the preference setting or be executed in a “safe” manner, akin to the human instinct for survival. To realize the set preferences, the control system relies on a management level that supervises the lower-level functions (which are the coordination and execution levels of the system) to ensure that the system is still on track toward its goals. The management level both supervises the lower-level functions and provides integrated information to the supreme system (or supervisor) to allow the generation of goals and assessment of system capabilities. The execution level of the autonomous control system comprises simple control algorithms and low-level numeric signal processing that can automatically conduct the routine work. The execution level is the lowest level that directly interacts with the environment and is thus usually built with sensors and actuators. Furthermore, a coordination level exists as an intermediary between the management and execution levels. Although the coordination level has lower decision-making authority than the management level, it can support the tuning, scheduling, and supervision of the execution level. By aiding preprocessing and simplifying the data that are derived from the execution level, the coordination level filters and clarifies the critical factor for the management level. Such is the architecture of an autonomous controller with interfaces to sense, actuation, human input, and other systems. For more details about the hierarchical structure of autonomous systems, please refer to [17,38].

### 3.2. Autonomy Level

The autonomy level guides the development of autonomous systems. From a mere machine to an autonomous one, the system has evolved toward a human-like control system. However, just like any accomplishment in research, the realization of an autonomous control system is a gradual and stepwise process, where each step is defined by the modeling of an autonomy level. Previous studies have proposed several autonomy levels for evaluating a system. Some researchers have formulated the levels of autonomy using biological concepts such as *autopoiesis* and recursive self-maintenance; Reference [26] provides a comparative survey and discussion. Because the autonomous control system is a replacement for human labor, its higher level of autonomy entails a lower degree of human intervention. One paper proposed the concept of using the level of independence from human input (which can be measured with the metrics of interaction time and frequencies as well as operator workload) as a part of grading standards [50]. In addition, because an autonomous control system must address environmental uncertainties, another paper [51] focuses on the dynamic of the environment, specifically, on how a system must scale up the autonomy level along with the aggressiveness of a system’s environment. Finally, recalling that the autonomous control system is a tool that is used to complete some set of goals, as discussed in Section 2.1.3, the complexity of the mission that is assigned also factors into the ranking of an autonomous control system. Thus, the autonomy levels of a particular control system are relative to the complexity of its assigned mission. Another autonomy classification is from the Society of Automotive Engineers (SAE), formulated with the intention to provide a public standard [52]. From level 0 of a nonautonomous vehicle to level 5 of a fully autonomous vehicle, SAE’s standard helps governments and automotive engineers more concisely define what an autonomous vehicle is.

In general, there are three aspects to the assessment of a system’s autonomy level: The extent of operator intervention, environmental difficulties, and the complexity of the mission. The relative standards for these three factors on evaluating the level of autonomy has been proposed in [53], which constructed a set of standard definitions that support a set of metrics.

## 4. Integration with Modern Computing Techniques

Thus far, we have only presented integration between the different levels of an autonomous system, such as the sensor, actuator, and control system, and the use of machine learning. This integration is only used for small-scale integration in autonomous systems. For better performance, autonomous systems can also be integrated with other modern technologies such as IoT, big data, and federated learning.

### 4.1. Internet of Things

**Figure 4:**
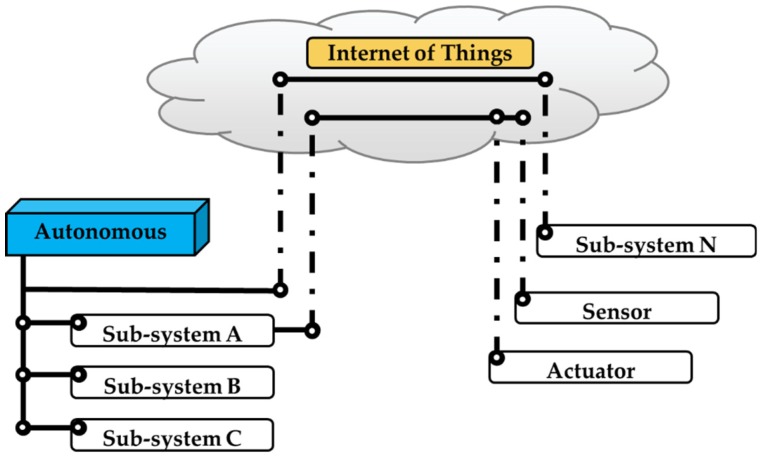
How IoT extends an Autonomous System.

IoT provides each machine the ability to automatically and adaptively sense, interconnect, and interact with each other. With IoT capabilities, the sensor can transmit the required data back to the autonomous system, and the actuator can respond according to system specifications. Specifically, in terms of the fundamental structure illustrated in Figure 3, machines with IoT capabilities can be considered an extension of the subsystem, actuator, and sensor of an autonomous system. Similar to how human beings use various tools, IoT extensions aid autonomous systems by extending its reach. Figure 4 illustrates how IoT greatly enlarges an autonomous system’s operating area.

IoT comprises the four major components of sensing, heterogeneous access, information processing, as well as security and privacy applications and services [54]. Having the same components as IoT, the implementation of Wireless Sensor Networks (WSNs), Machine to Machine (M2M), and Cyber Physical System (CPS) are also able to boost the performance of autonomous systems. WSNs can be used to monitor the physical and environmental conditions through adaptive sensor networks and a distributed autonomous sensor [55]. These sensors aid the WSNs in organizing the networks under a scarcity of resources such as power, computation, and storage. Similarly, M2M allows both wireless and wired systems to communicate with other devices of the same ability, and CPS can tighten the combination of and coordination between the system’s computational and physical elements. For more specific definitions of IoT, WSN, M2M, and CPS, please refer to [56,57].

### 4.2. Big Data

**Figure 5:**
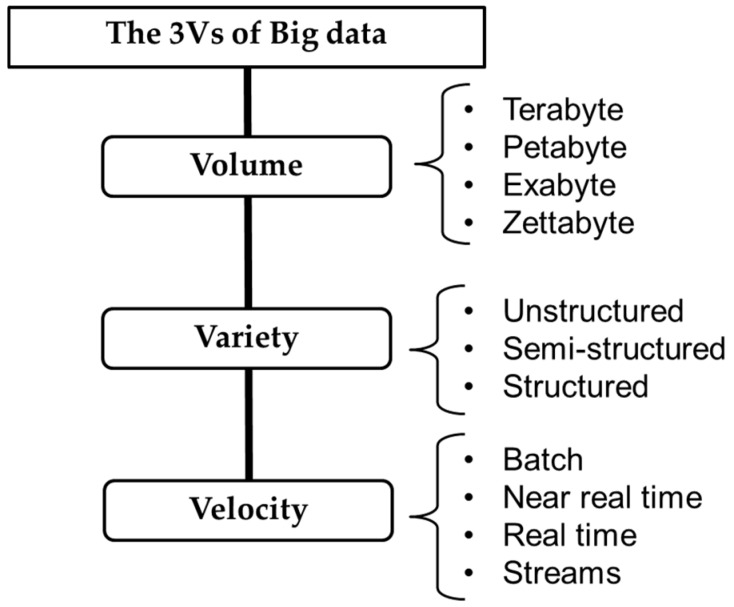
The three “Vs” of big data characteristics [58].

Big data involve large or complex data sets. Data of this scale cannot be adequately processed by traditional applications with regards to the three characteristics of volume, variety, and velocity (the three Vs; Figure 5). In [58], a brief description of the three Vs is provided, and a more detailed description is provided in [59]. Volume means the size of the data—which is of the scale of terabytes and petabytes, much larger than those of traditional storage devices. The variety of structured and unstructured data not only provides wide-ranging information but also raises difficulties on how knowledge can be extracted. Intelligent communication surmounts such difficulties [60] by using further contextual data, from sensors and image recognition technology, to define the user’s current environment. A data set with more data cases (rows) offers greater statistical power, and a more complex data set (with more attributes or columns) leads to a higher false discovery rate. The velocity of big data, from batch to real-time to streams, is required for different time-limited processes. With the arrival of the era of 5G network densification [61], the velocity of data (from loading to striding to streaming) will only increase. The analysis and visualization of big data aid in the extraction of hidden patterns and correlations among the data. Some big data processing platforms that are supported by analytical tools can extract information from complex, dynamic environments; these platforms can support decision-making by giving recommendations and automatically detecting anomalies [62].

However, despite all the advantages of big data, security is still a major concern. For example, the original data directly received from the sensor are considered the explicit data when building a model. Such explicit data are often classified as confidential (or private if the model was used on a personal device). To solve this issue, federated learning should be used in implementations of big data.

### 4.3. Federated Learning and Over-the-Air Technology

**Figure 6:**
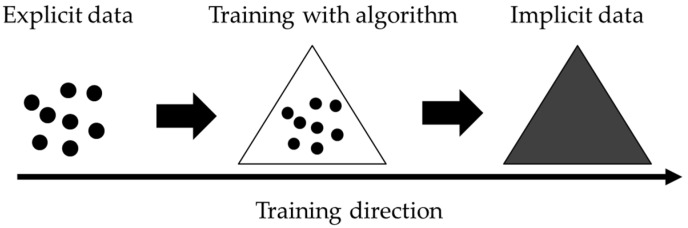
Explicit and Implicit data. Implicit data are embedded in the algorithms, and explicit data are separated from the algorithms.

Federated learning is a distributed machine learning approach that enables model training with large amounts of decentralized data. Initially, the local models are individually trained with the decentralized data. Subsequently, each locally trained model is assembled by the central system. The system would then optimize the model and send the supreme model back to the local device. Unlike traditional machine learning algorithms that are designed for highly stable and controlled environments, federated learning can handle unreliable and relatively slow network connections [63]. Given the wide operating range of autonomous systems, federated learning should be adopted. Moreover, federated learning only shares the implicit data but not the origin (or explicit) data, as depicted in Figure 6; the relations between implicit and explicit data are also illustrated in Figure 6. Federated learning is highly prized by users who prioritize confidentiality and privacy. By only sharing the model (implicit data), the confidentiality of businesses and individuals is assured.

**Figure 7:**
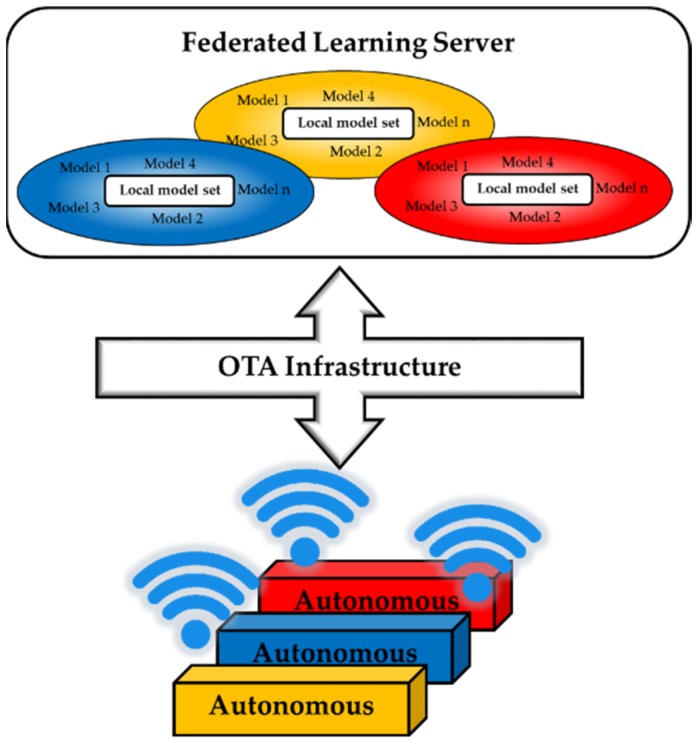
The system sends models Over-the-Air for federated learning.

Similar to the design of structures of federated learning on mobile devices [64], autonomous systems must also be integrated with an updating system. A leading updating program is Over-the-Air (OTA) technology. OTA not only allows a wireless device to upgrade software securely but also makes the device able to transmit data back to the data center (Figure 7). OTA has already been widely used on mobile phones, and it is adopted by advanced automobiles to reduce recall expense, hasten recall compliance, and improve cybersecurity responses. Another application of OTA as an update mechanism is illustrated in [65].

### 4.4. Integration of Autonomous Systems

**Figure 8:**
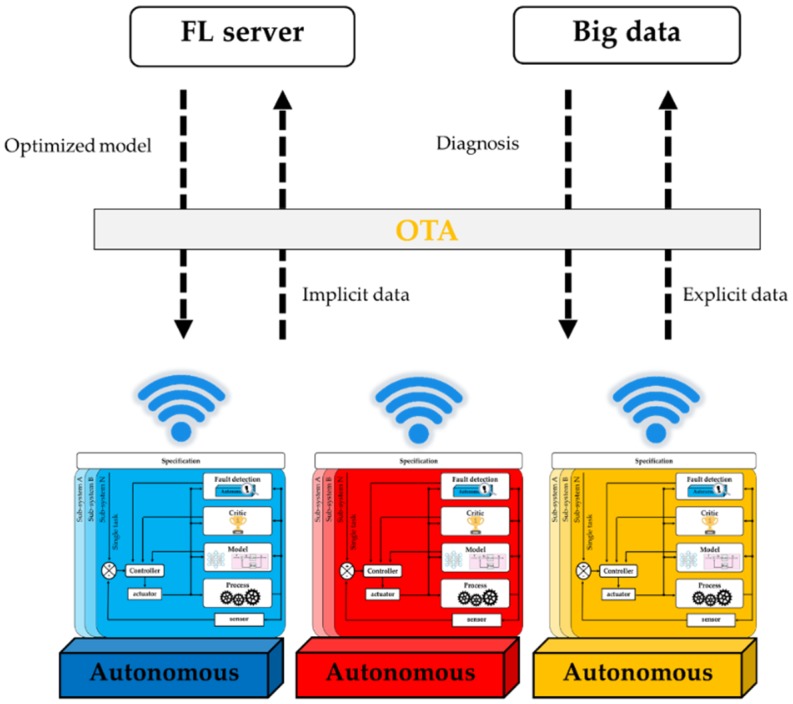
Integration of autonomous systems.

Interdisciplinary efforts are required to construct autonomous systems that can solve problems like a human being, where each development in a related discipline leads us closer toward the goal. Figure 8 illustrates the integration of an autonomous system into a system. Specifically, the autonomous system can extend its working area using IoT as the communicative interface. A federated learning server combines the model (implicit data) generated from autonomous systems to construct the optimized model. Although such use in conjunction with big data technologies can increase the false discovery rate, hidden patterns and correlations among the data can be extracted for the fault detection function in the autonomous system.

## 5. Autonomous Systems Vehicles

Autonomous cars can operate without human steering and pedaling. A modern autonomous vehicle can sense its surrounding, classify objects that it detects, and interpret sensory information to navigate itself in accordance with transportation rules [24]. Historically, the first autonomous car was radio controlled made by Houdina Radio Control Company in New York City [66]. The car received radio impulses sent by the other car that was in front of it. Such impulses steered the electric steering motors connected to the wheel, thus steering the car. Subsequently, in 1953, the RCA Labs built a self-propelled car that was guided by wires laid under the floor. This idea so fascinated the state traffic engineer of Nebraska that he convinced RCA researchers to jointly implement the concept to vehicles (with the state) to reduce accidents from driver error and fatigue [67,68]. At that time, researchers were still navigating autonomous vehicles through external mechanisms. In the 1980s, the vision-guided driverless robotic van was designed by Dickmanns and his team, which reached speeds of over 60 km/h under no-traffic conditions [69]. In 1995, the first self-steering autonomous driving car used neural networks to successfully drive over 5,000 km across the United States [70]. Developments in autonomous vehicles have proceeded in conjunction with developments in sensor technology. Modern sensors used in autonomous vehicles primarily involve cameras, lidar, radar, sonar, global positioning systems, inertial measurement units, and wheel odometry. Sensors provide the input data for the inner program of the autonomous vehicle to analyze to control the steer, brake, and speed of the vehicle [71]. The use of autonomous vehicles can lower the number of traffic accidents caused by human beings. Currently, some have predicted that by 2035, most cars would be operated with no human intervention. However, some have expressed opposition to autonomous vehicles [25]. Critics cite worries over more expensive vehicles due to the integration of modern technology, complications from attributing responsibility in accidents caused by the malfunction of autonomous vehicles, compromises to security and privacy from hacking or sharing of tracking data. Transportation professionals must strike a balance between the costs and benefits of autonomous vehicles.

## 6. Autonomous Systems in Health Monitoring

An autonomous system is a control system that integrates various modern techniques. With the aforementioned integrations in Section 2 and Section 5, our proposed structure of autonomous systems can be used in medical fields for monitoring anesthesia induction. The monitoring of anesthesia is crucial to surgery. Reliable and steady readings on the anesthesia index help doctors to better decide whether to start surgery. Presently, the anesthesiologist is still relied on to maintain the patient’s level of anesthesia by reducing or increasing the dosage of the anesthetic. Fortunately, equipment such as the Bispectral Index (BIS) monitor [72] predicts a patient’s awareness during anesthesia induction. This greatly reduces the burden on the anesthesiologist, who now only needs to adjust the dosage of the anesthetic drug according to the given index (in addition to attending to emergencies during the surgery).

Despite the convenience of BIS equipment, it cannot address environmental uncertainties such as interference from the surgical instrument or a detachment of the electrode patch. Moreover, the absence of a feedback mechanism that allows the machine to autonomously maintain the patient’s level of awareness during surgery (by maintaining the dosage of the drug) causes its inability to have a higher level of autonomy. We are eager to surpass such a limitation and thus propose our autonomous system structure that is integrable with modern techniques. In our proposal, first, the vital signs are collected through embedded sensors. Subsequently, through IoT techniques, the collected data are sent to the systems. With feature extraction algorithms that are based on domain knowledge (e.g., the continuous wavelet transforms and short-time Fourier transforms) [73], the data are preprocessed. After preprocessing, the data can be trained by the selected algorithm for obtaining the suitable model. While the model is being optimized by the critic function, the system self-organizes the predicting strategy, that is, whether to use the current prediction method or to switch to another vital sign signal–based prediction method due to the interferences in the previous type of data. The fault detection function can use the embedded sensors to detect disturbances caused by the detachment or incorrect placement of body sensors [74]. Finally, with the feedback mechanism, the autonomous system can be used for the automated control of the patient’s level of anesthesia based on the computed evaluations. Furthermore, the models and data that are generated at every surgery can be gathered with OTA techniques. These models and data can be used to improve federated learning techniques and big data predictions. The data derived from the sensor can also be transmitted through IoT, which allows the model function to be adopted and updated with AI algorithms. In addition, the critic function can be executed with federated learning, and faults can be detected, diagnosed, and predicted with big data analysis through OTA techniques.

## 7. Discussion

Machines were invented to perform boring and repetitive jobs. In the 18th century, the steam engine brought about the industrial revolution, and machine power has replaced animal and human power. Today, AI will bring a revolution similar to the revolution bought by the steam engine. As AI develops, it will outperform the human brain. Tasks, such as product inspection, classification, and identification, once considered so complex that only a human being can perform them are now gradually being executed by machines. Precise and needing no rest, machines will eventually outperform humans in certain tasks. Researchers are dedicated to designing autonomous systems that are capable of adapting to various operational conditions without human intervention. However, even if an autonomous system has full autonomy, a human being must still assign its mission. This mission is planned according to human needs, and human needs are derived from human desire. This fundamental disparity between people and machines lies in the fact that human beings possess consciousness.

Whether people have consciousness was originally a philosophical debate. However, since the ultimate goal of machines is to acquire human-like behavior, this debate is reprised in the field of engineering. Although some researchers believe that the possession of consciousness is a goal to pursue [75], others warn that we should avoid it [76]. Attempts to implement consciousness into machines are also being discussed in [77]. Consciousness, particularly human desire, drives advances in civilization. By contrast, we believe that machines have no consciousness and thus cannot think. Because machines have no desire apart from fulfilling their assigned mission, they only care about the error associated with the defined value; no matter how complicated the mechanism for the machine’s fulfilment of its missions, it can always be parsed into practical mechanisms. Conversely, if machines can be made conscious, does this not mean that human decisions are guided by some unknown mechanism? The day when machines reveal desires of their own is the day when they have reached the ultimate level of autonomy.

## 8. Conclusions

From steam engines to supercomputers, humans have been searching for a replacement to ourselves. Initially, machines substituted muscle. Currently, the computer is gradually substituting the brain. Even if machines cannot think, machines can definitely learn. Thus, we believe that machines can be taught to think. A field that aims toward human-like machine behavior is the field of autonomous systems.

Aiming to establish the capacity for self-governance into machines, the topic of autonomy in engineering is popular in both hardware and software applications. Focusing on the system’s ability to independently adapt to uncertainty, engineers have constructed hierarchy levels for coordination within the system and autonomy levels for comparisons between the abilities of individual autonomous systems. Hierarchy levels that evolve with the mechanism inside the autonomous system are essential to machines in continuous operation. Subsequently, with the model, the system can construct a knowledge of the environment that they are interacting with. Furthermore, with the critic function, the adopted model is guaranteed to be optimal. Parts of the system exhibiting signs of malfunction are excluded through the fault detection function. The highest level is the specification that is in charge of task distribution. The specification supervises the entire system to ensure it is on track to its assigned mission. Furthermore, to test its ability to imitate human problem solving, the autonomous system is graded by the extent of necessary operator intervention. Such tests ensure that the machine can be independent, where environmental difficulties in addition to the complexity of the mission test the system’s limits under uncertainty. Both hierarchy and autonomy levels guide the development of systems toward full autonomy; hierarchy levels allow researchers to know which function to implement, and autonomy levels allow researchers to know the next step to reach.

Finally, human beings have the intelligence to use and invent tools, including machines. Because tools can be improved upon, autonomous systems can extend its ability through integration with other modern techniques such as IoT, big data, OTA, machine learning, federated learning, and control systems. Because the effort toward autonomous systems are interdisciplinary, the autonomy structure seems to be a combination of many functional aspects of technical systems in fields such as intelligent control, machine learning, IoT, and big data. Developments and applications in these fields typically focus on a single function and are seldom integrated with each other toward system autonomy. Thus, integrated systems have not been realized: Complex, high-level solutions only solve high-level problems, and the low level only deals with basic problems. Nonetheless, autonomous systems are likely to be realized in the foreseeable future.
